# Correlation between EGFR gene mutation, cytologic tumor markers, 18F-FDG uptake in non-small cell lung cancer

**DOI:** 10.1186/s12885-016-2251-z

**Published:** 2016-03-16

**Authors:** Arthur Cho, Jin Hur, Yong Wha Moon, Sae Rom Hong, Young Joo Suh, Yun Jung Kim, Dong Jin Im, Yoo Jin Hong, Hye-Jeong Lee, Young Jin Kim, Hyo Sup Shim, Jae Seok Lee, Joo-Hang Kim, Byoung Wook Choi

**Affiliations:** Department of Nuclear Medicine, Severance Hospital, Yonsei University College of Medicine, Seoul, Korea; Department of Radiology and Research Institute of Radiological Science, Severance Hospital, Yonsei University College of Medicine, Seoul, Korea; Yonsei Cancer Center, Division of Medical Oncology, Department of Internal Medicine, Yonsei University College of Medicine, Seoul, Korea; Medical Oncology, Department of Internal Medicine, CHA Bundang Medical Center, CHA University, Seongnam, Korea; Department of Pathology, Severance Hospital, Yonsei University College of Medicine, Seoul, Korea; Department of Pathology, Dongguk University Ilsan Hospital, Dongguk University College of Medicine, Goyang, Korea; Department of Radiology, Severance Hospital, Yonsei University College of Medicine, 50 Yonsei-ro, Seodaemun-gu, Seoul, 120-752 Korea

**Keywords:** EGFR mutation, Cytologic CYFRA 21-1, Cytologic tumor marker, 18F-FDG PET/CT

## Abstract

**Background:**

EGFR mutation-induced cell proliferation causes changes in tumor biology and tumor metabolism, which may reflect tumor marker concentration and 18F-FDG uptake on PET/CT. Direct aspirates of primary lung tumors contain different concentrations of tumor markers than serum tumor markers, and may correlate better with EGFR mutation than serum tumor markers.

The purpose of this study is to investigate an association between cytologic tumor markers and FDG uptake with EGFR mutation status in non-small cell lung cancer (NSCLC).

**Methods:**

We prospectively collected tumor aspirates of 61 patients who underwent EGFR mutation analysis. Serum and cytologic CYFRA 21-1, CEA, and SCCA levels were measured and correlated with EGFR gene mutations. FDG PET/CT was performed on 58 patients for NSCLC staging, and SUV was correlated with EGFR mutation status.

**Results:**

Thirty (50 %) patients had EGFR mutation and 57 patients had adenocarcinoma subtype. Univariate analysis showed that female gender, never smoker, high levels of cytologic CYFRA 21-1 (c-CYFRA) and lower maximum standard uptake value (SUVmax) were correlated with EGFR mutations. ROC generated cut-off values of 20.8 ng/ml for c-CYFRA and SUVmax of 9.6 showed highest sensitivity for EGFR mutation detection. Multivariate analysis revealed that female gender [hazard ratio (HR): 18.15, *p* = 0.025], higher levels of c-CYFRA (HR: 7.58, and lower SUVmax (HR: 0.08, *p* = 0.005) were predictive of harboring EGFR mutation.

**Conclusions:**

The cytologic tumor marker c-CYFRA was positively associated with EGFR mutations in NSCLC. EGFR mutation-positive NSCLCs have relatively lower glycolysis compared with NSCLCs without EGFR mutation.

## Background

Discovery of certain epidermal growth factor receptor (EGFR) mutations that affect tyrosine kinase inhibitor (TKI) efficacy in non-small cell lung carcinoma (NSCLC) has increased the importance of identifying patients harboring EGFR mutations. TKIs have been shown to prolong progression-free survival, and activating EGFR mutations have been shown to predict the response to EGFR-TKI therapy [1–3]. Sociopathological factors predicting EGFR mutations are Asian descent, female gender, and never-smokers; however, patient selection for EGFR mutation analysis cannot be made on these factors alone [2].

Serum tumor markers such as carcinoembryonic antigen (CEA), squamous cell carcinoma antigen (SCCA), and cytokeratin 19 fragments (CYFRA 21-1) are clinically used for NSCLC screening and recurrence evaluation, and some of these tumor markers have been shown to be correlated with prognostic factors such as higher TNM stages [4, 5]. Although none of these tumor markers have been correlated with EGFR mutation status, CYFRA 21-1 has been shown to be useful in the prediction of TKI response in EGFR mutation patients, suggesting a potential correlation between CYFRA 21-1 and EGFR mutation status [6, 7].

Recently, we have shown that tumor markers derived from cytologic fluid aspirated from the primary lung cancer site can be clinically useful in the differential diagnosis of lung cancer and NSCLC subtyping [8–10]. We have shown that there is a weak correlation between cytologic tumor markers and their serum counterparts, which suggests an additional mechanism for their release into the serum. Although no studies thus far have shown a correlation between serum tumor marker levels and EGFR mutation status, in vitro studies using hepatocellular carcinoma and head and neck cancer cell lines have shown an EGF-dependent increase in cytokeratin 19 [11, 12].

Increased fluorine-18-flurodeoxyglucose (FDG) uptake in lung cancers has been shown to be a prognostic factor for NSCLC patients, and lower SUV has been reported to be associated with favorable outcomes in EGFR targeted therapy [13]. These findings suggest a potential correlation between FDG uptake with EGFR mutation and serum tumor markers. However, the correlation between FDG uptake with EGFR mutation have not been satisfactorily evaluated.

Therefore, we conducted this study to prospectively investigate an association between cytologic tumor markers and FDG uptake with EGFR mutation status in NSCLC.

## Methods

### Patient selection

**Fig. 1:**
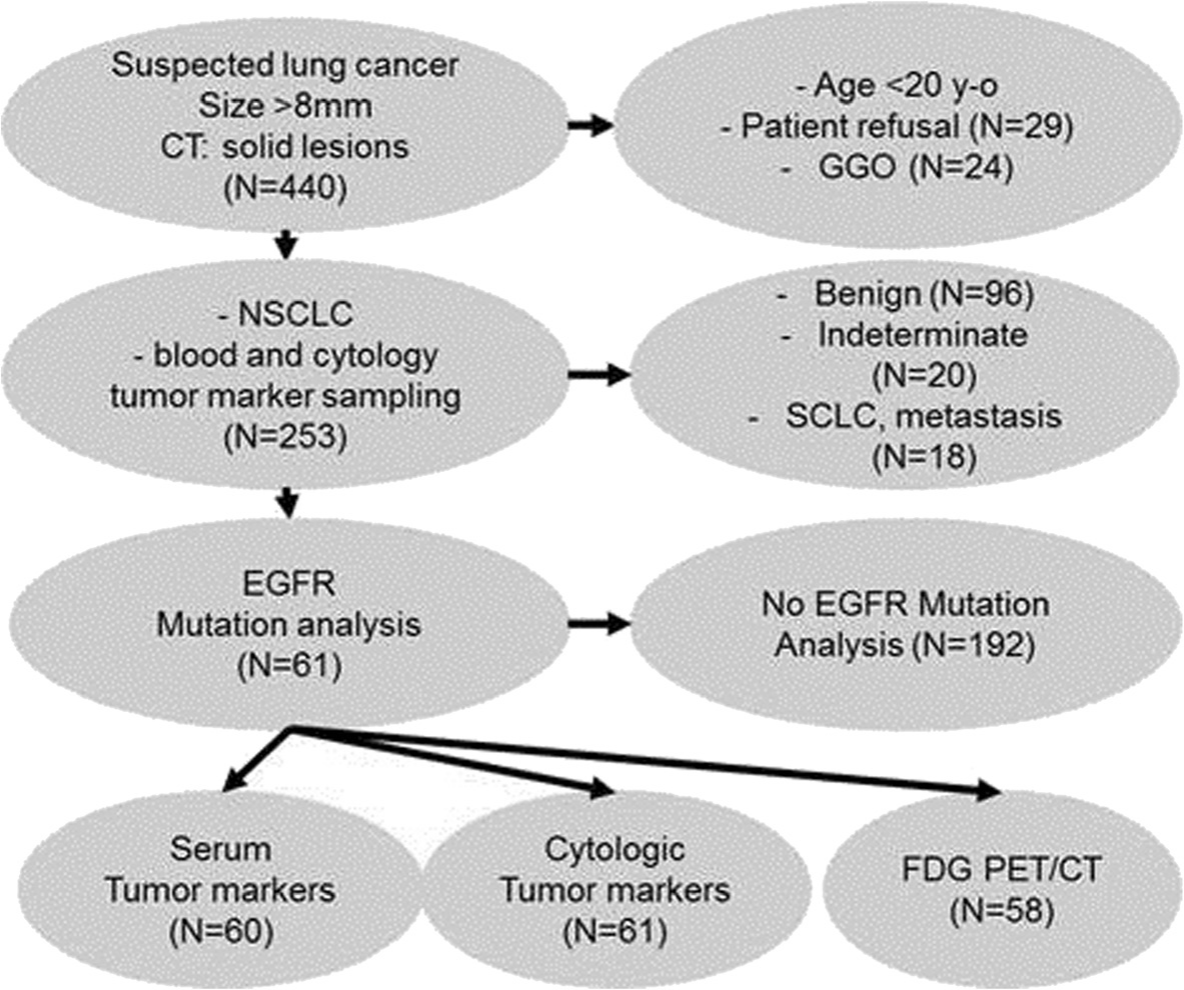
Study design and patient selection algorithm

From April 2009 to November 2012, a total of 440 patients suspected of primary lung malignancy were prospectively enrolled for fine-needle aspiration biopsy (FNAB) of lung nodules or masses and to evaluate cytologic tumor marker levels in needle aspirates. The inclusion criteria were age greater than 20 years, lesion size more than 8 mm, and solid lesions (ground glass opacity component less than 50 %). The exclusion criteria were ground glass opacity lesions (*n* = 24) and refusal to provide informed written consent (*n* = 29). Of these 440 patients, 253 had NSCLC pathology, 96 had benign lesions, 18 had metastasis or small cell lung cancer pathology, and 20 had indeterminate results. To test the application of cytologic tumor markers in the prediction of EGFR mutations, only patients (*n* = 253) with lesions pathologically confirmed to be NSCLC were included. Finally, 61 patients were included (33 men and 28 women; average age 61 ± 10 years) who underwent EGFR mutation analysis and had pathologically confirmed NSCLC (Fig. 1).

The charts of these 61 patients were reviewed to evaluate for age, gender, smoking history, pack year, and TNM staging. Never-smoker was defined as less than patients who smoked less than 100 cigarettes in their lifetime or patients who stopped smoking for more than 15 years prior to the study and who smoked less than ten packs of cigarettes per year. Former smoker was defined as more than 3 months of smoking cessation before lung cancer diagnosis. fluorine-18-flurodeoxyglucose positron emission tomography/computed tomography (FDG PET/CT) was performed in the staging workup of NSCLC to evaluate for N- and M-staging. Of the 61 patients, semi-quantitative FDG uptake evaluation was not possible in three patients due to the PET/CT having been obtained at other hospitals. All other FDG PET/CT scans were obtained before surgery or further treatment. Data collection was systematized and a standardized registration form was prepared. This study was approved by the institutional ethics committee of Yonsei University College of Medicine, and all patients provided informed written consent.

### Percutaneous transthoracic needle aspiration biopsy technique

The biopsy procedures were performed by three experienced chest radiologists, who had more than 4 years of experience in performing thoracic biopsies. Fluoroscopy-guided biopsy interventions (*n* = 21) and CT-guided biopsy interventions (*n* = 40) were performed using previously stated methods [9]. Briefly, one needle puncture was used to obtain at least two aspiration specimens which were separated into two components for cytological examination and cell block processing. Remaining aspirates (1–2 cc) were rinsed with 1 mL of normal saline solution in a tube for the evaluation of cytological tumor markers.

### Tumor marker analysis

Tumor markers analyzed were serum CYFRA 21-1 (s-CYFRA), serum CEA (s-CEA), serum SCCA (s-SCCA), cytologic CYFRA 21-1 (c-CYFRA), cytologic CEA (c-CEA), and cytologic SCCA (c-SCCA). All serum samples and cytological fluid aspirates were collected prior to any therapy. Serum and cytological fluid analyses were performed using the same methods as previously described [9]. Serum samples were obtained within 1 or 2 days of FNAB. Cytological fluid samples were assayed twice for tumor marker levels, and the mean values were used for analysis. Detectable levels for each cytological fluid tumor marker were defined as follows: 0.1–500 ng/ml for CYFRA 21-1 and CEA, and 0.01–150 ng/ml for SCCA.

### PET/CT protocol and imaging analysis

All patients underwent routine FDG PET/CT scans with either Discovery 600 PET/CT (GE Healthcare, Milwaukee, WI, USA) or Biograph TruePoint 40 PET/CT (Siemens Medical Systems, CTI, Knoxville, TN, USA). All patients fasted for at least 6 h and glucose levels in peripheral blood in all patients were confirmed to be 140 mg/dl or less before FDG injection. Approximately 5.5 MBq/kg of FDG was administered intravenously 1 h before image acquisition. After the initial low-dose CT (Discovery 600: 30 mA, 130 kVp; Biograph TruePoint: 36 mA, 120 kVp), standard PET imaging was performed from the skull base to the proximal thighs with an acquisition time of 3 min/bed in three-dimensional mode. Images were then reconstructed using the ordered subset expectation maximization algorithm (two iterations, 20 subsets).

All PET/CT images were reviewed by an experienced nuclear medicine specialist on one GE AW 4.0 workstation (GE Healthcare, Milwaukee, WI, USA). Identification of the aspirated primary lesion was done by reviewing images obtained during biopsy. On PET scans, a volume of interest (VOI) was drawn on the primary lesion. The maximum standard uptake value (SUVmax) of the primary lesion was then obtained and recorded. Total lesion glycolysis (TLG) of the primary lesion was also obtained by using an isocontour of 40 %; if the VOI was out of proportion to the lesion seen on CT, either a cut-off SUV of 2.5 was used or the threshold was adjusted to best fit the contour of the lesion on CT. TLG was calculated by the multiplication of volume (cm^3^) with the mean SUV within the VOI.

### EGFR mutation analysis

Genetic analysis was performed to determine activating EGFR mutations in exon 19, exon 20, or exon 21. The nucleotide sequence of the kinase domain of the EGFR gene, from exon 18 to 21, was determined using nested polymerase chain reaction amplification of the individual exons, as previously described [14, 15].

### Statistical analysis

EGFR mutation status was used as a reference standard for analysis. Categorical variables (smoking status, gender, TNM staging, and pathology) were analyzed by either the chi-squared test or the Fisher’s exact test. Continuous variables (age, serum and cytologic tumor marker levels, SUVmax, and TLG) were first evaluated for normality using the Kolmogorov-Smirnov test, and *p*-values > 0.05 were assumed to fulfill the normality assumption. Due to the wide distribution of TLG values (range: 1.1–787.8), natural logarithmic transformations were applied to TLG (renamed to log(TLG)) to obtain normally distributed data (fulfill the Kolmogorov-Smirnov assumption). Parametric analyses was used for normally distributed variables, otherwise non-parametric analyses were used. The Student’s *t*-test was used to compare average values of tumor marker and FDG parameters according to EGFR status.

Receiver operating characteristic (ROC) curves were constructed using the continuous variables that showed significant differences in the average values on *t*-test. A cut-off value was determined for the highest sensitivity for predicting EGFR mutations. A logistic regression model was then used to evaluate the association between clinicopathologic factors with an EGFR mutation. Statistically significant findings on univariate analysis were used in the multivariate analysis. *P* values of less than 0.05 were considered statistically significant. All statistical analyses were carried out using SAS (version 9.2, SAS Inc., Cary, NC, USA), with the exception of Medcalc (version 9.5, MedCalc, Mariakerke, Belgium) which was used for the ROC analysis.

## Results

### Patient demographics

A total of 61 patients (28 female, mean age 61 years) were included in this study. EGFR mutations were identified in 30 patients (49.1 %), of which most had either an exon 19 deletion (*n* = 18), exon 21 mutation (*n* = 10), or exon 20 mutation (*n* = 2). A significantly higher portion of female distribution (20 of 30 patients, *p* = 0.001) and never smokers (21 out of 30, *p* = 0.007) were seen among patients harboring an EGFR mutation. The major pathology of the lesions was adenocarcinoma (*n* = 58), whereas two patients had squamous cell pathology and one patient had NSCLC not otherwise specified. The TNM stage in this study population was nearly equally distributed, with 26 patients at TNM stage I/II, and 35 patients at TNM stage III/IV. There was no significant difference in TNM staging between the patients harboring the different EGFR mutations. Patient demographics are shown in Table 1.

**Table 1:** Patient characteristics

Variable	Wild-type EGFR	EGFR mutation	*p*-value
Age	63.1 ± 7.8	60.3 ± 11.8	0.282
Gender (F:M)	8:23	20:10	0.001^a^
Pathology (ADC:SCC:NOS)	28:1:1	29:1:0	0.611
Smoking status			0.007^a^
Never smoker	11	21	
Current/former smoker	20	9	
TNM staging (1:2:3:4)	8:7:5:11	7:4:5:14	0.746
Tumor markers			
s-CYFRA	7.28 ± 20.28	2.50 ± 1.67	0.201
s-CEA	18.77 ± 46.91	34.61 ± 67.55	0.300
s-SCCA	1.00 ± 1.03	0.84 ± 0.89	0.507
c-CYFRA	85.18 ± 135.79	200.19 ± 208.84	0.014^a^
c-CEA	21.60 ± 59.05	49.85 ± 110.04	0.214
c-SCCA	9.97 ± 35.36	5.70 ± 19.33	0.562
SUVmax	10.33 ± 5.82	7.01 ± 3.91	0.014^a^
Log(TLG)	1.80 ± 0.65	1.35 ± 0.70	0.014^a^

*ADC* adenocarcinoma, *c-CEA* cytologic carcinoembryonic antigen, *c-CYFRA* cytologic CYFRA 21-1, *c-SCCA* cytologic squamous cell carcinoma antigen, *NOS* non-small cell carcinoma not otherwise specified, *SCC* squamous cell carcinoma, *s-CEA* serum carcinoembyonic antigen, *s-CYFRA* serum CYFRA 21-1, *s-SCCA* serum squamous cell carcinoma antigen, *SUVmax* maximum standard uptake value, *log(TLG)* natural logarithmic transformation of total lesion glycolysis

^a^statistically significant values of *p* < 0.05

### EGFR status and tumor marker levels

There was no significant difference in serum tumor marker levels between wild-type and mutant EGFR (7.3 ng/ml vs 2.5 ng/ml for s-CYFRA, 18.8 ng/ml vs 34.6 ng/ml for s-CEA, and 1.0 ng/ml vs 0.8 ng/ml for s-SCCA). In regards to cytologic tumor markers, only c-CYFRA was significantly different between EGFR status, with higher levels of c-CYFRA with EGFR mutations compared with wild-type EGFR (200.2 ng/ml ± 208.8 ng/ml vs 85.2 ng/ml ±135.8 ng/ml, *p* = 0.014). Other cytologic tumor markers showed no significant differences between EGFR status (21.6 ng/ml vs 50 ng/ml for c-CEA and 10 ng/ml vs 5.7 ng/ml for c-SCCA) (Table 1).

**Fig. 2:**
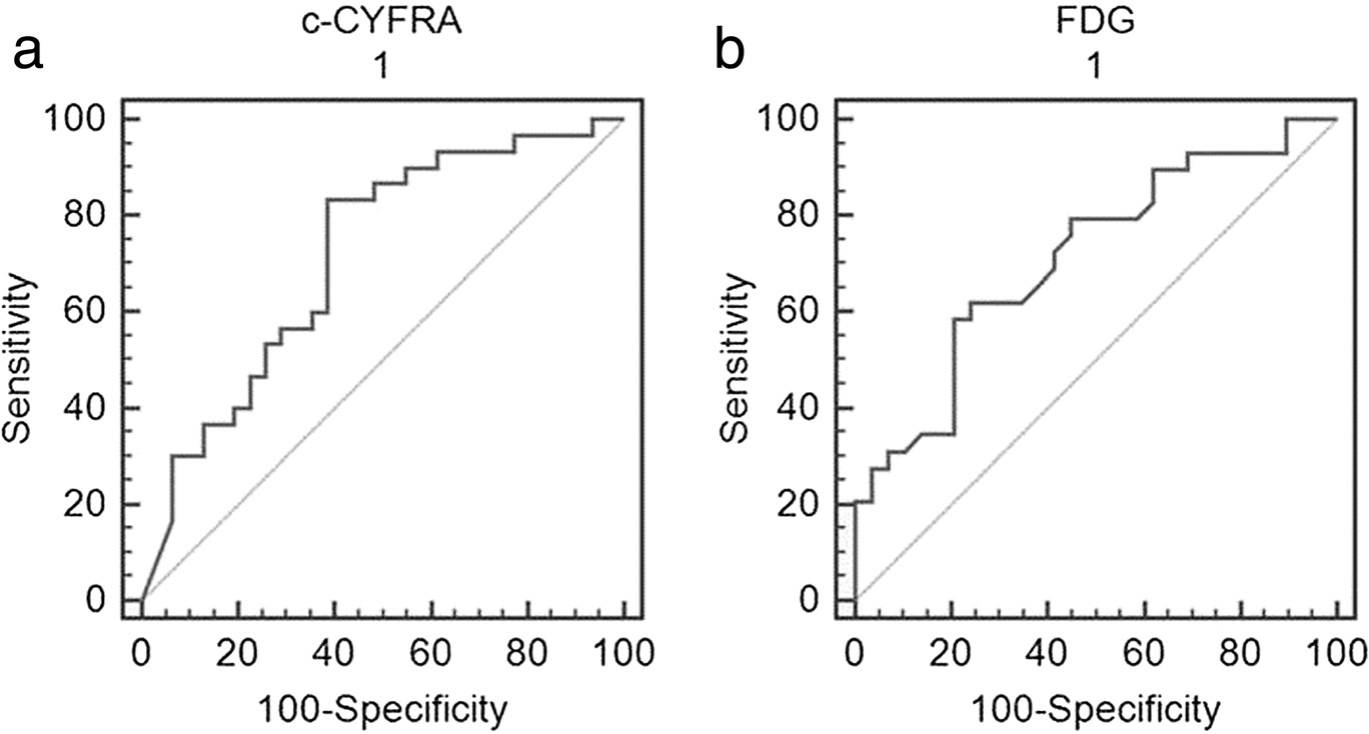
Receiver operating characteristic (ROC) curves of c-CYFRA level and FDG uptake (SUVmax) in predicting EGFR mutations. **a** ROC of c-CYFRA showed that a cut-off of 20.8 ng/ml had the highest sensitivity of 83.3 % (area under the ROC curve = 0.715, *p* = 0.001) in discriminating EGFR mutations from wild-type EGFR. **b** ROC of FDG uptake showed that a SUVmax cut-off of 9.6 had the highest sensitivity of 79.3 % in predicting EGFR mutations (area under the ROC curve = 0.68, *p* = 0.010)

ROC analysis evaluating the c-CYFRA cut-off value that best predicts EGFR mutations resulted in 20.8 ng/ml for the highest sensitivity of 83.3 % [95 % confidence interval (CI): 65.3–94.3, area under the curve (AUC) = 0.715, *p* = 0.001) (Fig. 2a). Using this cut-off, 18 out of 23 patients (78 %) with low c-CYFRA levels were wild-type EGFR, and 25 out of 38 patients (65.8 %) with high c-CYFRA levels had mutant EGFR, resulting in an overall specificity of 58.1 % and an overall accuracy of 70.5 %.

### EGFR status and PET/CT results

**Fig. 3:**
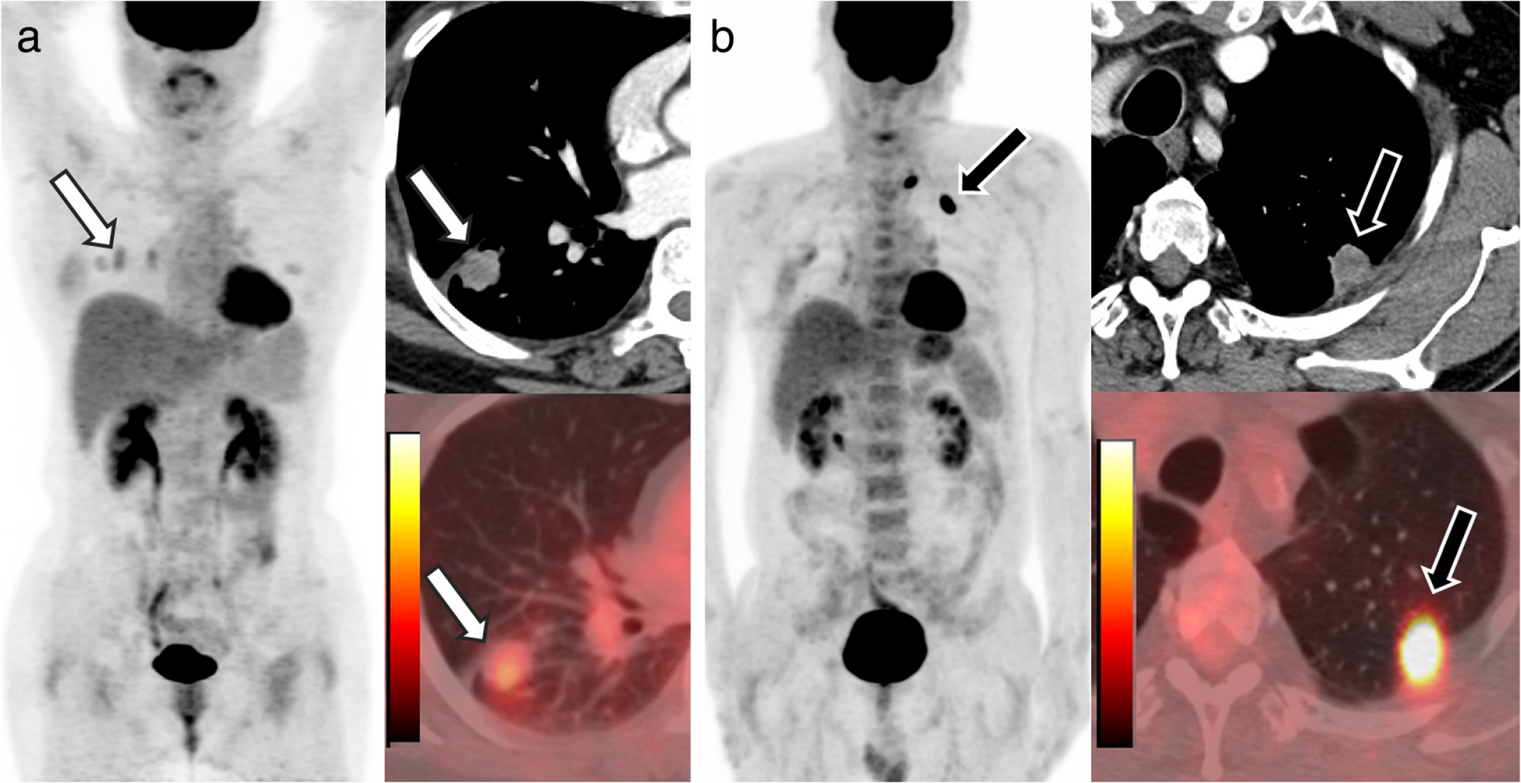
Representative figures of differences in FDG uptake according to EGFR status. **a** A 55 yo female with 21 mm sized adenocarcinoma in the right lower lung superior segment. SUVmax was 2.2, and TLG was 4.0. **b** A 58 yo female with 20 mm sized adenocarcinoma in the left upper lung apical segment. SUVmax was 14.6, and TLG was 31.5

The average SUVmax was significantly lower in EGFR-mutated lesions compared to wild-type (7.0 ± 3.9 vs 10.3 ± 5.8, *p* = 0.014), and log(TLG) was lower in EGFR-mutated lesions (1.80 ± 0.65 vs 1.35 ± 0.70, *p* = 0.014). Figure 3 shows representative images of patients with 18F-FDG uptake according to EGFR status. The correlation between c-CYFRA and FDG uptake using Pearson correlation showed no significant correlation between the two (0.001 for SUVmax, *p* = 0.996, and -0.049 for log(TLG), *p* = 0.79).

ROC analysis resulted in 9.6 as the cut-off for SUVmax with the highest sensitivity for predicting an EGFR mutation (sensitivity 79.3, 95 % CI: 60–92, AUC = 0.68, *p* = 0.010) (Fig. 2b). Using this cut-off, 23 out of 29 patients (79.3 %) with EGFR mutations had low FDG uptake, and 15 out of 29 patients (51.7 %) had high FDG uptake in EGFR wild-types, with an overall accuracy of 65.5 %. For log(TLG), a cut-off of 1.64 showed highest sensitivity in predicting EGFR mutation. Using this cut-off, compared to SUVmax, log(TLG) showed lower sensitivity (72.4 %) and slightly increased accuracy (69.0 %) in predicting EGFR mutation.

### Multivariate analysis in predicting EGFR mutation

Significant variables predicting EGFR mutations were gender, smoking status, c-CYFRA, SUVmax, and log(TLG). These variables were used in the multivariate analysis for predicting an EGFR mutation (Table 2). Two models were used due to multicollinearity between SUVmax and log(TLG). Of these variables, female gender [hazard ratio (HR): 18.15, 95 % CI: 1.44–228.7, *p* = 0.025], higher levels of c-CYFRA (HR: 7.58, 95 % CI: 1.57–36.61, *p* = 0.012), lower FDG uptake (HR: 13.00, 95 % CI: 2.20–76.62, *p* = 0.005), and lower log(TLG) (HR: 24.15, 95 % CI: 0–0.38, *p* = 0.005) were significantly correlated with EGFR mutations.

**Table 2:** Multivariate analysis in the prediction of EGFR mutation

	Model 1		Model 2	
	HR (95 % CI)	*P* value	HR (95 % CI)	*P* value
Gender	18.15 (1.44–228.73)	0.025^a^	39.69 (2.12–741.68)	0.014^a^
Smoking status	1.44 (0.16–13.24)	0.749	1.58 (0.17–15.02)	0.692
c-CYFRA^b^	7.58 (1.57–36.61)	0.012^a^	6.82 (0.03–0.77)	0.023^a^
SUVmax^c^	12.97 (2.20–76.62)	0.005^a^		
Log(TLG)^d^			24.15 (0–0.38)	0.005^a^

*c-CYFRA* cytologic CYFRA 21-1, *HR* hazard ratio, *SUVmax* maximum standard uptake value

^a^statistically significant values of *p* < 0.05

^b^c-CYFRA cut-off value: 20.8 ng/ml

^c^SUVmax cut-off value: 9.6

^d^Log(TLG) cut-off value: 1.64

## Discussion

We have shown a positive correlation between c-CYFRA and EGFR mutation status in predominately solid-type NSCLC. C-CYFRA levels higher than 20.8 ng/ml have 83.3 % sensitivity and 70.5 % accuracy in predicting EGFR mutation status. In addition, we have also shown that these solid type NSCLCs showed lower FDG uptake (7.0 ± 3.9) in EGFR mutation-positive lesions compared with EGFR wild-type (10.3 ± 5.8).

In our study, we evaluated three widely used serum tumor markers in NSCLC and their cytologic counterparts to evaluate for EGFR mutation prediction. We found that c-CYFRA levels correlated with EGFR mutation status. CYFRA 21-1 is cytokeratin 19 fragment (CK 19), a member of the intermediate filament protein family, which contributes to the mechanical integrity of the cell and participates in cell division, motility, and cell-to-cell contact [16]. EGFR stimulation activates a signaling cascade, promoting cell division, migration, angiogenesis, and apoptosis inhibition, which suggests an increase in structural cytokeratins. This has been confirmed in cell studies whereby levels of CK 19, [11, 12] CK 6, and CK 16 [16] have been increased in response to EGF stimulation. We have shown that activating EGFR mutations are correlated with increased CK 19 expression in human lung cancers.

In accordance with previous studies, there was no correlation between s-CYFRA levels and EGFR mutation status. This may be explained by the proposed mechanisms of c-CYFRA release into the serum. Dohmoto et al. and others showed CK 19 fragment release into the serum is related to tumor necrosis and cell death mechanisms such as cleaving enzymes, caspase-3, and apoptosis [17–20]. This may explain the lack of evidence for correlation between s-CYFRA and EGFR mutation, as activated EGFR induces apoptosis inhibition which has been a proposed mechanism for CYFRA 21-1 release into the serum. Additionally, the half-life of cytokeratin fragments in the circulation is about 10–15 h [16], which adds to an additional time variation in the correlation between s-CYFRA levels in patients with an EGFR mutation. We have clinically shown in a previous study that s-CYFRA is only weakly correlated with c-CYFRA levels [8], which may also be corroborating evidence for this proposed mechanism.

The correlation between EGFR mutation status and FDG uptake has not been well-established. Previously, two studies found no statistical difference in FDG uptake between EGFR mutation and wild-type EGFR [21, 22]. Chung et al. also used metabolic tumor volume and SUVmax in their analysis, but did not find a significant correlation between SUVmax and EGFR mutation status (wild-type SUVmax 9.1 vs mutation SUVmax 8.6) [22]. In contrast, three studies have found a positive correlation, but it is not established which EGFR mutation is correlated with higher FDG uptake. Moreover, two studies showed a positive correlation with lower FDG uptake in EGFR wild-type as compared with mutant EGFR [23, 24]. Na et al. suggested that using a SUVmax cut-off of 9.2, 40 % was wild type EGFR, but only 11 % were EGFR mutation. Mak et al., as also suggested similar trend in that SUVmax normalized to blood showed that EGFR mutation showed lower FDG uptake compared to wild type EGFR (2.9 vs 3.4, respectively). In contrast, one study found higher FDG uptake in the EGFR-mutated NSCLCs (SUVmax 10.5) compared with wild-type (SUVmax 8.0) [25]. In our study, we have shown that SUVmax was significantly lower with EGFR mutation status compared with wild-type, and TLG was lower as well. The major difference between this study and previous reports is the homogeneity of the lesions. Due to the prospective nature of this study in using FNAB, we selected lesions that were predominately solid and larger than 8 mm, which incidentally reduces SUV-related artifacts such as partial volume effects found in bronchoalveolar subtype adenocarcinomas and in lesions smaller than 5 mm. We suggest that these conditions resulted in a more reliable estimate of higher glucose metabolism in wild-type EGFR compared with mutant EGFR. Previous reports have shown that FDG PET/CT may be helpful in predicting responses to TKI therapy [26–28], and the combination of FDG PET/CT, c-CYFRA, and EGFR mutation status may be helpful in TKI treatment selection and therapy response.

Recently, lung cancer in female gender and never smokers has received considerable attention with the advent of EGFR TKI treatment. Studies have shown that patients with no smoking history, female sex, adenocarcinoma histologic type, Asian ethnicity, or EGFR mutations are predictive of EGFR TKI treatment [29]. In accordance with previous studies, we have shown in our study a higher distribution of female gender (67 %) and never smokers (70 %) in EGFR mutant patients. Among these clinicopathologic factors, mutations in the EGFR gene have been shown to have the strongest predictive power. Due to the added benefit of increased progression-free survival when using TKI in EGFR mutation-positive patients, there has been an increasing importance in predicting EGFR mutations. Multiple randomized phase III trials on TKI treatment compared to cisplatin based chemotherapy regimens have shown significant survival benefit of TKI for patients with EGFR mutation. However, patients with EGFR wild-type showed significant survival benefit with cisplatin based therapy, which further places emphasis on the clinical importance of EGFR mutation analysis [30–32]. We have shown in our results a high sensitivity in predicting EGFR mutation when using c-CYFRA levels. Evaluation of c-CYFRA levels is as rapid as s-CYFRA, which may provide clinicians with an additional quick and sensitive method to anticipate which patient may harbor EGFR mutation.

Our study has some limitations. First, only a relatively small number of patients were evaluated for EGFR mutation analysis. Only 61 out of 253 patients had an EGFR mutation, which can be a potential selection bias. However, it is often unfeasible for clinicians to use EGFR mutational analysis in all patients. In our study, 49 % of patients had an EGFR mutation, which was similar to the reported higher incidence of EGFR mutations in Asians. A recent meta-analysis showed that EGFR mutations in Asians with adenocarcinoma pathology is 47.9 % (1492 out of 3117 patients) [33], which is much higher than the reported incidence in European patients (10 %) [2, 34], Second, the results may be influenced by cut-off point selection for cytological tumor markers. There are no normal reference values for tumor marker concentrations in cytological fluid. In our study, we used ROC curves to determinate the cut-off values of tumor markers in the cytological fluid. Third, we did not evaluate EGFR-TKI treatment response and survival analysis according to c-CYFRA levels.

## Conclusions

The cytologic tumor marker c-CYFRA was positively associated with EGFR mutations in NSCLC. C-CYFRA levels, higher than 20.8 ng/ml, have 83.3 % sensitivity and 70.5 % accuracy in predicting EGFR mutation status. In addition, we have also shown that EGFR mutation has relatively lower glycolysis compared with wild-type EGFR. Higher levels of c-CYFRA may reflect the cellular changes associated with activating EGFR mutation, and further studies are needed to evaluate for the additional benefit of including c-CYFRA and FDG uptake in EGFR-targeted therapy evaluation.

## Abbreviations

18F-FDG: fluorine-18-fluorodeoxyglucose
CEA: carcinoembryonic antigen
CYFRA 21-1: cytokeratin 19 fragments
EGFR: epidermal growth factor receptor
FNAB: fine-needle aspiration biopsy
PET/CT: positron emission tomography/computed tomography
ROC: receiver operating characteristic
SCCA: squamous cell carcinoma antigen
SUVmax: maximum standard uptake value
TLG: total lesion glycolysis
VOI: volume of interest
